# Navigating infant food insecurity: low-income parents infant feeding intentions and practices in the UK

**DOI:** 10.1186/s12889-025-25822-2

**Published:** 2025-12-08

**Authors:** Emma Hunter, Flora Douglas

**Affiliations:** School of Health, Ishbel Gordon Building, Robert Gordon University, Garthdee, Aberdeen, AB10 7QE UK

**Keywords:** Food insecurity, Infant feeding, Infant feeding practices, New parents, Health inequalities, Wider determinants of health, Lived experience, Interviews, Qualitative

## Abstract

Food insecurity is increasingly being recognised as a public health issue in high income countries, exacerbated for many by the recent cost-of-living crisis. High food prices mean many households, especially those with children, are vulnerable to food insecurity. Since 2021, and the emergence of the UK’s ‘cost-of-living crisis’, food charities have reported increased requests for support from families with young children. However, the experience of food insecurity amongst families with very young infants remains poorly understood. To the best of our knowledge, this is the first qualitative study exploring UK parents’ and carers’ lived experiences of the impact of the cost of living crisis on their infant feeding intentions and practices. Semi-structured interviews (*N* = 11) were conducted with parents’/ carers’ living in Scotland, who self-identified as living with food insecurity and were or had been responsible for feeding an infant aged 0–6 months born any time from January 2022. Using thematic analysis, seven main themes were identified: 1) The struggle to afford food and formula in the face of reduced income, 2) Earning but struggling: a lack of financial support for parents in work, 3) Hard work and sacrifice, parents' actions to secure food for their baby, 4) Don’t ask, don’t tell: limited to no conversations about finances or formula with healthcare professionals, 5) Positive views about breastfeeding but challenges dictating practice, 6) Income shortfall struggles: the provision of support from family and third sector organisations, 7) Experiences of stigma and guilt accessing and receiving support. Parents can struggle to afford food to feed their families and infant formula, which could lead them to sacrifice their own food needs. Families and third sector organisations often provide financial and instrumental support for new parents living on a low income. However, this study indicates that some parents can experience stigma and embarrassment accessing this type of support. Conversations with healthcare professionals around new parent’s financial circumstances and infant formula could provide an opportunity for intervention and support.

**Supplementary Information:**

The online version contains supplementary material available at 10.1186/s12889-025-25822-2.

## Introduction

Food insecurity is defined as a ‘lack [of] regular access to enough safe and nutritious food for normal growth and development and an active healthy life’ [1]. Food insecurity is increasingly being recognised as a public health issue in high income countries (BMC [2]). It is estimated that globally, 6.3% of the population in high income countries is experiencing food insecurity [3]. Food insecurity has been exacerbated for many by the recent cost-of-living crisis [4–6]. The United Kingdom (UK ‘cost of living crisis’ refers to a fall in real disposable incomes (i.e., adjusted for inflation and after taxes and benefits in relation to the rising cost of living,a situation that the UK has experienced since late 2021 [7]. Within this period, there have been sharp increases in the price of healthy, nutritious food and it is estimated, that to eat in line with UK Government healthy eating recommendations, as set out in the Eatwell Guide [8], those in the most deprived fifth of the population would need to spend 45% of their disposable income on food, rising to 70% in households with children [9]. This highlights how households with children may be more vulnerable to experiencing food insecurity compared to those without [9]. Financial challenges placed on new families and women specifically, may also contribute to food insecurity.

Women may be more likely to experience food insecurity, compared to men, for a number of reasons. For example, for women in work, maternity leave and the move to receive statutory maternity pay often means women and their families experience a significant drop in average earnings that can lead to financial hardship [10]. Additionally, women are more likely than men to work part time, be employed in a low-paid position, have reduced time to work due to higher levels of unpaid domestic and caring work and head a single parent household [11–13]. As a result, they are often more reliant on benefits [14]. Furthermore, UK Government fiscal policy such as the two-child limit, where government financial assistance (i.e., Universal Credit and Child Tax Credit) is only available for a maximum of two children within the household, has meant many families, and women and children in particular, are pushed further into poverty [14, 15].

Across the UK, third sector organisations have seen increasing numbers of families utilising their services, for example, the Trussell Trust, an anti-poverty charity and food bank network operating within the UK, reported 508,000 of 1,428,000 food bank parcels (63%) distributed between April and September 2024 went to families with children aged 0–16 years [16]. Furthermore, in the UK, there are over 300 baby banks, voluntary organisations providing essential items including clothing, nappies, toys and equipment such as prams to families, many of whom reported referral rates increasing by 54.4% between 2021 and 2023 [17].

Schemes such as Best Start in Scotland and Healthy Start in England, Wales and Northern Ireland provide financial assistance to enable low income families to purchase healthy food (fruit, vegetables, pulses), milk and infant formula [18, 19]. However, Healthy Start payments were last uprated in 2021 and there has been no recent increase, despite rising food price inflation during the cost of living crisis [20] placing increasing pressure on the budgets of low income families. Additionally, the price of infant formula increased by 18–36%, depending on the brand, between December 2021 and December 2023 [21], further stretching the budgets of families who chose to formula feed.

In keeping with the commitment made by the Scottish Government to eradicate the existence and need for food banks, many Health Boards across Scotland look to refer new parents struggling to afford food and/or formula, to a cash first response in the first instance [22–24]. The cash-first approach not only helps people access financial capital, i.e. through the provision of money or shopping vouchers, it also provides monetary advice and support around accessing benefits or support available from relevant organisations within the community [25].

While food insecurity has been tracked at the household level and work undertaken to tackle child food poverty and secure the right to food for children in the UK through policy development [26], food insecurity prevalence amongst pregnant women and families with infants under one is not currently assessed and remains poorly understood in the UK. Despite growing attention to child food insecurity, there is a lack of data on families with very young infants. Given the continued cost of living crisis, the reduced financial circumstances often faced by new parents and the high cost of infant formula, there is now an urgent need for research to identify key priorities and policy actions required to mitigate household food insecurity experience at this crucial life stage.

Funded by the Transforming the UK Food Systems (TUKFS) Programme, established in 2020 to provide and assess evidence to tackle UK food system challenges, supported by investment from the UKRI’s Strategic Priorities Fund [27], this research was undertaken as part of the Diet and Health Inequalities (DIO Food) project [28]. The DIO Food project aims to connect with vulnerable populations facing diet and health inequalities (early years and people living on a low income), alongside retailers, to explore experiences and gather data to generate timely responses to inform policy and practice [28]. Health inequalities exist as a result of wider economic, social, political and environmental circumstances or determinants and policies to tackle health inequalities need to reflect such factors in order to be effective [29]. To ensure proper consideration of these wider determinants, that impact both mental and physical health [29], a health equity approach, which frames health in the context of economic, social and environmental determinants [30] was adopted. This approach helped focus the interview questions and consider the arising data. As one of the first studies on this topic, it is hoped this exploration of infant food insecurity, will contribute to the development of effective, evidence-based, equitable future policies and practices.

The key aim of the current study was to capture parents’ and/or carers’ lived experiences and explore influences that they feel impact or impacted infant feeding, associated with the cost of living crisis. We looked to explore this through the following objectives:

1. Investigate food and feeding-related challenges parents and families have experienced during the first six months of their infants’ lives
2. Explore parents/carers’ experiences of realising (or otherwise) their feeding intentions during their infant’s first six months
3. Investigate parents/carers’ perceptions of factors that help or hinder their infant feeding experiences3

## Methods

### Advisory group

Keeping in mind the different health professionals and support organisations with which new parents living on a low income may interact, an Advisory Group, comprised of expert members from 3rd sector organisations (anti-poverty organisations and charities supporting families), the healthcare sector (UK, NHS) and academia was established and met regularly throughout the project. The Advisory Group guided us throughout the study design, delivery, analysis and dissemination phases. 

### Participants and setting

We recruited parents or carers who were, or who had been, responsible for feeding a baby aged 0–6 months (born any time from January 2022; when the cost-of-living crisis was established and widely recognised in the UK), who self-reported being food-insecure and were aged 18 or over. A screening questionnaire which included a two item food insecurity screener [31], was developed to determine an individual’s eligibility to participate in the study (Supplementary data 1).

**Fig. 1:**
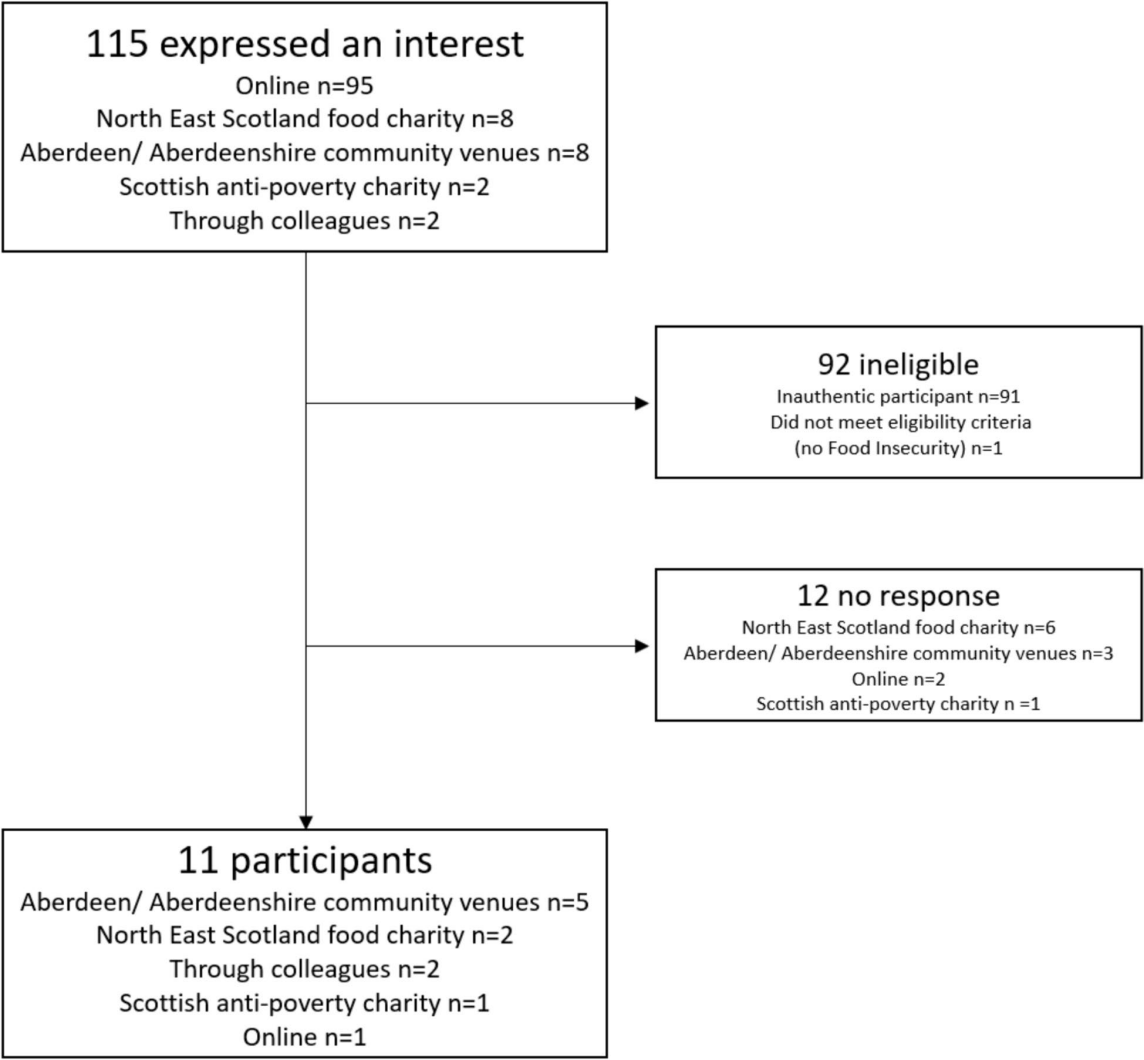
Flow chart of participant recruitment

Following discussions with our Advisory Group members, who agreed this method of recruitment would be acceptable and relevant, and to help enable and encourage participation of parents who had not previously disclosed their financial challenges to third sector organisations or service providers, study information was shared on social media platforms Facebook and X. Unexpectedly, the social media posts resulted in a large number of expressions of interest (Fig. 1). However, interviews with two participants, where neither participant was willing or able to put on their camera, did not include discussions around topics we would typically expect when discussing antenatal care in the UK context (no mention of the GP, midwife or health visitor), and instead focused on hospital visits and high costs of healthcare. Upon reflection, discussion with the wider research team and colleagues, and a review of the existing literature, it was agreed that we had likely been contacted by imposter or inauthentic participants (full details: 10.31219/osf.io/gzfxh_v1). The threat to data integrity led us to pause online recruitment and revise our study protocol. Revisions included amending the inclusion criteria to state that participants wishing to meet over Teams would need to attend an online screening call with their camera turned on for at least part of the meeting and the removal of information regarding monetary recompense for participation from recruitment leaflets. Following these amendments, only one participant was recruited to the study through this recruitment stream. This experience led the research team to focus on the targeted approach to recruitment through trusted organisations and existing networks which had been implemented alongside the online recruitment stream. Our advisory group members were provided with study information, including, where requested, physical recruitment fliers, to allow for dissemination with any organisations or colleagues they felt could help with recruitment or with parents utilising their services. During meetings with our advisory group, members who indicated an awareness of eligible parents using their services, expressed confidence that these parents would be willing to talk with us. Other group members stated they knew of organisations or services attended by eligible parents with whom they could circulate study information. Advisory group members and the research team agreed that recruiting participants through individuals and organisations with which the parents were already familiar and with whom they had built trust, would be an appropriate and acceptable pathway for this participant group. E.H. and F.D. also undertook extensive engagement within the local community and with third sector and Government organisations. However, despite reassurance from those working with and experienced in supporting parents and carers living on a low income, including our Advisory Group members, who informed us that food insecurity was an issue faced by those attending services they provided and therefore, would easily be able to help with recruitment, this was not the case and recruitment proved challenging.

Potential participants were provided an information sheet either in person or via email. Eligibility was assessed either in person where the individual attended a mother and baby/toddler group visited by E.H., or through a brief Teams call where participants were required to turn on their camera for at least five minutes to deter any further inauthentic participants or the possibility of any individual participating multiple times. The screening questionnaire was administered verbally during these meetings. Once eligibility had been established, participants were invited to take part in an interview either on Teams or over the telephone. Before the interview commenced, verbal, informed consent was obtained and recorded for each participant.

Ethical approval for the study, in accordance with the declaration of Helsinki [32], was sought and obtained from the Robert Gordon University School of Nursing, Midwifery and Paramedic Practice School Ethics Review Panel (SERP reference number 23–09, approved on 21 st December 2023).

### Data collection

A brief screening questionnaire (Supplementary data 1), initially developed to be delivered online and then latterly utilised during a face to face discussion either in person or on Teams, collected information including age range, gender, ethnicity, food insecurity status and current financial situation (optional). The two item food insecurity questionnaire, developed by Hager et al. [31], was selected due to its ability to identify households with children at risk of food insecurity and its convergent validity with more commonly used, longer and more burdensome screeners, such as the Household Food Security Survey. The screening questionnaire also confirmed the individual was or had been responsible for feeding a baby aged 0–6 months.

Participants were invited to take part in semi-structured interviews, at a time that was convenient for them, using Teams, over telephone or in person (where the participant was within reasonable travelling distance for the research team), depending on their preference. An interview topic guide, developed for this study and designed to be used flexibility, helped steer the discussion whilst allowing for follow up questions on topics not identified by the research team (Supplementary data 2). This allowed for an inductive approach to the generation of themes and sub themes.

Before consent was sought, participants were informed that they did not have to answer any questions with which they were uncomfortable and that they could take a break at any point. Participants were also informed of their right to withdraw without consequence. Questions were phrased sensitively given the difficult subject matter and had been reviewed by our expert Advisory Group for appropriateness and acceptability. All interviews were audio recorded using a digital audio recorder (TASCAM DR-07X). Participants were each provided a £25 retail gift voucher for giving up their time and sharing their expertise.

### Data analysis

All participants were assigned a participant identification number. The file linking participant names with these numbers was password protected and stored within a separate electronic folder to that which contained the interview data. All audio recordings were transcribed verbatim by a University approved data transcription service (Red Balloon). E.H. checked all transcripts for accuracy and removed any sensitive or potentially identifiable information, such as names or any geographical locations mentioned during discussions, to maintain participant anonymity. Thematic analysis of the interview transcripts was undertaken by two researchers (E.H., F.D.), in line with steps outlined by Braun and Clake [33]. After familiarisation with the data (E.H., F.D.) initial descriptive codes were generated (E.H.) which were then reviewed (F.D.) and discussed (E.H., F.D.). Emerging themes were shared and discussed with the advisory group and the wider DIO Food team. This process continued until all themes and sub themes were finalised. A summary of the themes and sub themes, in an accessible eZine format, was shared with participants to gather their feedback. NVivo 14 software was used to support and manage the data analysis.

Using a health equity lens to consider the economic, social and environmental determinants impacting feeding decisions and practices and ultimately the health of infants and their parents, we explored parents experiences of breast and/or formula feeding their baby while living on a low income.

### Reflexivity

Qualitative research is undertaken by individuals situated within a specific context, time and space. It is argued, this must be acknowledged and that researchers should reflect and highlight their situatedness to allow transparency of the factors that could play a role on the questions asked, the data collected and the interpretation of this data [34]. In the current study, in depth interviews were primarily conducted by E.H., a white woman, mother and research fellow in food insecurity and obesity. E.H., and F.D. (a white woman, mother and Professor in Public Health) analysed the data. While E.H. has lived and raised her children in areas classified as ‘highly deprived’ and F.D. has extensive insight into maternal food insecurity stemming from her experiences as a nurse and continuing through her academic research, neither E.H. or F.D. has personally experienced food insecurity. The Advisory Group helped ensure the feasibility and acceptability of the research proposed, the study design and the questions under investigation. However, while E.H. sought to build rapport with participants and create space for them to openly share their experiences and both E.H. and F.D. aimed to interpret participants’ accounts without judgment or comparison to their own feeding decisions or practices, the authors lived experience and knowledge on the topic will have undoubtedly shaped data collection, interpretation and analysis.

## Results

### Participants

Participants (*N* = 11) were recruited over a period of ten months. The majority of participants were recruited from mother and baby groups held at community venues in Aberdeen and Aberdeenshire (*n* = 5). Participants were also recruited from third sector organisations including a North East food charity (*n* = 2) and a Scottish anti-poverty charity (*n* = 1), through colleagues within Robert Gordon University (*n* = 2) and online (*n* = 1) (Fig. 1). Most were female (*n* = 10, 90.1%) aged between 25–34 (*n* = 6, 54.5%) and White ethnicity (*n* = 10, 90.1%). All participants resided in Scotland, with the majority living in urban areas (*n* = 8, 72.7%). There were four single parent families consisting of mum and baby (*n* = 3) and mum, baby and a teenage child (*n* = 1), six families of three: mum, dad and baby, and one family of four: mum, dad, baby and older child. The majority of participants indicated they had worried their food would run out before they could afford to buy more (*n* = 8, 72.7%) while the remaining participants reported actually running out of food and being unable to afford to purchase more. Most participants indicated they were receiving or had been receiving statutory maternity pay during the first six months of their infants life (*n* = 7, 63.3%). Just over half the participants fed their baby as they had intended (*n* = 6, 54.5%) (Table 1). However, four participants who intended to breastfeed did not end up doing so due to issues including a lack of support, breastfeeding ‘just not working’, baby seeming distressed when feeding, and anxiety around milk production and baby not getting sufficient milk (although none of these reasons were described as being directly linked with food insecurity by the parent).

**Table 1:** Frequency table summarising participant demographic data, food insecurity status, income source, and feeding intentions and practices

		n (%)
Gender	Female	10 (90.9%)
	Male	1 (9.1%)
Age range	18–24	3 (27.3%)
	25–34	6 (54.5%)
	35–44	2 (18.2%)
Ethnicity	White (British, Scottish, English, Irish)	10 (90.9%)
	Asian	1 (9.1%)
Food Insecurity Status	Within the past 12 months I/we worried whether our food would run out before we got money to buy more	8 (72.7%)
	Within the past 12 months the food I/we bought just didn't last and we didn't have money to get more	3 (27.3%)
Income source of participant during infants first 6 months	Benefits	3 (27.3%)
	Statutory Maternity Pay	7 (63.6%)
	Part time work/Student	1 (9.1%)
Feeding intention	Breastfeeding	6 (54.5%)
	Formula feeding	4 (36.4%)
	Combi feeding	1 (9.1%)
Feeding practice	Breastfeeding	1 (9.1%)
	Formula feeding	8 (72.7%)
	Combi feeding	2 (18.2%)
Fed in line with intention	Yes	6 (54.5%)
	No	5 (45.5%)

Interviews, conducted between February and June 2024, were held online using Teams (*n* = 4), over the phone (*n* = 6) or in person (*n* = 1) and lasted between 22 and 44 min.

### Thematic analysis

Thematic analysis of the interview data revealed seven main themes and 11 sub themes (Table 2). Four main themes and seven sub themes were generated in relation to Objective 1, one main theme and two sub themes in relation to Objective 2 and two themes and two subthemes in relation to Objective 3.

**Table 2:** Summary of the main themes and sub themes

Themes	Sub themes
Objective 1: Investigating food and feeding-related challenges parents and families have experienced during the first 6 months of their infants’ lives	
1. The struggle to afford food and formula in the face of reduced income	i) More mouths to feed, benefits and maternity pay don’t cover outgoings leaving limited funds for food ii) High cost of infant formula
2. Earning but struggling: a lack of financial support for parents in work	
3. Hard work and sacrifice, parents' actions to secure food for their baby	i) Cutting back or going without food to ensure baby is fed ii) Using strategies and multiple resources to maximise food budget
4. Don’t ask, don’t tell: limited to no conversations about finances or formula with healthcare professionals	i) No discussion around financial circumstances ii) Strained conversations around infant formula iii) Trial and error and sources of advice on suitable formula
Objective 2: Exploring parents experiences of realising (or otherwise) their feeding intentions during their infant’s first 6 months	
5. Positive views about breastfeeding but challenges dictate practice in reality	i) Best for health, bonding and budget friendly, the perceived benefits of breastfeeding ii) Didn’t go as planned… challenges of breastfeeding
Objective 3: Investigating parents’ perceptions of factors that help or hinder their infant feeding experiences	
6. Income shortfall struggles: the provision of instrumental support from family and third sector organisations	i) Covering costs, the role of family ii) Support in the community (3rd sector organisations)
7. Parent experiences of stigma and guilt accessing and receiving support	

## Objective 1: Investigating food and feeding-related challenges parents and families have experienced during the first 6 months of their infants’ lives

### Theme 1

#### The struggle to afford food and formula in the face of reduced income

All participants spoke of the struggle to afford infant formula and food for themselves. The majority of participants in the current study were receiving statutory maternity pay which often represented a significant drop in income. Others were relying on Government financial assistance. Within the context of a reduced income, soaring food and energy prices linked to the cost-of-living crisis and the high cost of infant formula, participants described struggling to cover their outgoings.

#### More mouths to feed, benefits and maternity pay don’t cover outgoings leaving limited funds for food

All participants spoke about the difficulty continuing to cover their outgoings on a limited budget. Participant 01, who was in receipt of Universal Credit, talked to the (impossible) task of affording to pay all her bills, purchase everything she needed for her baby and find the money needed to feed herself. While the Government Best Start food card helped her purchase infant formula, with a tub sometimes lasting four or five days she was sometimes forced to rely on others to help her acquire the necessary amount in order to allow her to properly feed her baby:

*‘at the minute I can't. So, I'm on Universal Credit, which I get £488 a month for everything, you know, and that does not, I mean, once I've paid my bills, like I'm still using foodbanks... I have a Best Start food card which will maybe get me like two tubs of formula a month, maybe three, and apart from that I have to try and rely on friends and family to, you know, chip in to help me get the rest cause, you know, it just doesn't add up’* (Participant 01, female, formula feeding, single parent)

Another participant, who had moved to statutory maternity pay during her infants first six months, talked about rising food bills and the injustice of families having to continue to feed themselves plus another person on what is often a significantly reduced income:

*‘our food bills definitely did go up…it was expensive, particularly on statutory maternity pay. I think it's criminal that they expect people to be able to feed a new person but pay you way less than you would normally be getting’* (Participant 02, female, combi feeding, family of 4)

#### High cost of infant formula

The high cost of infant formula was a topic that frequently emerged as a point of discussion during the interviews. Parents whose children required non-standard formula milk, i.e., lactose free or anti-reflux products, experienced even greater costs than those using, already expensive, standard formula. Participant 03 likened this increased cost to being *‘punished for something you can't help’*. Parents also expressed concern about the continually rising costs of formula as their baby grew and consumed increasingly greater volumes:

*‘she's going from 4oz, which is four scoops, to 6 at the moment, so that's already 50% extra each one, so it's going to kind of exponentially get faster and faster that we go through it, which is going to get more and more expensive’* (Participant 03, male, formula feeding, family of 3).

### Theme 2

#### Earning but struggling: a lack of financial support for parents in work

Those who were not entitled to benefits to ‘top up’ their reduced income, due to reasons such as their partners income or being on maternity leave from paid employment and in receipt of maternity pay, expressed frustration at the lack of financial help and support for mid earners, something they felt was required in the face of the cost of living crisis.

*‘I am a bit bitter there's not, you know, most of the supports, and quite rightly so, are aimed at the, you know, the low income families in the area, but you do feel sometimes like there's a sort of middle group of people, not the high earners, not the non-earners, that are like kind of ignored in it but they'd still be struggling because of the cost of living essentially… everything's just gone up so much that you think, well, what, what support are you entitled to as somebody who's earning though’* (Participant 02, female, combi feeding, family of four)

### Theme 3

#### Hard work and sacrifice, parents' actions to secure food for their baby

Parents described how increased outgoings and the need to prioritise their baby’s needs ahead of their own, led them to sacrifice their own food intake. Sacrifices involved reducing food intake or even going without food altogether. In order to maximise their food budget, participants spoke of various (often effortful) strategies that they used to acquire food, formula and other essentials.

#### Cutting back or going without food to ensure baby is fed

Participant 10 described how the expense of infant formula, alongside other necessities, i.e., nappies and wipes, plus the increased energy costs associated with keeping their flat warm, all in the face of a drop in household income as she moved to receive statutory maternity pay, resulted in a significantly reduced food budget for her and her partner. This participant shared how they made drastic cuts to the food they bought and consumed:

*‘we were buying like cheap freezer foods and things like that, especially cause we didn't want to admit to people, to start with, that we were finding it a bit tough. So, we were maybe having one meal a day, erm we were just kind of saving ourselves to dinnertime’* (Participant 10, female, formula feeding, family of 3)

She spoke of the detrimental effects this sacrifice had on her mental wellbeing and their energy levels:

*‘it was kind of dark, darkish place for us, and my husband's job's quite physical as well, so he was getting tired all the time and he was kind of falling asleep when he'd come home from work, just a lack of energy and that was causing a bit of like friction between us because I felt like I wanted a bit of help, and he, he couldn't understand it’*

Ultimately, their financial struggles meant they were forced to give up their own flat and move in with one of her parents.

Another participant, a single mother of two children, living on Universal Credit after poor mental health resulted in her having to give up her job, described how she had ‘*gone days sometimes and not really eaten very much to make sure that my children have got food in their stomachs*’ (Participant 09, female, formula feeding, single parent with 2 children).

#### Using strategies and multiple resources to maximise food budget

Participants described ways they attempted to mitigate food and feeding related challenges, including the ‘work’ undertaken and the use of various strategies and resources in order to acquire food within their budget. Strategies included careful planning around where to shop for formula and baby items based on price, and shopping in multiple stores for these various items. Participant 08 talked to the time consuming nature of this practice but expressed her ‘luck’ that she could access all the stores she required on foot.

*‘So, I get her bottle, I get her milk from one place, her nappies from another place and her wipes from another place. Yeah, which takes a bit more time than just getting everything just in one place. I go to the shops a lot, but I'm lucky, the shops that I've found, it's all kind of walking distance, so, it's not so bad. It's just a lot of planning’* (Participant 08, female, formula feeding, family of three)

One participant, a volunteer at a local food pantry and a food pantry user themselves, also described taking actions such as using supermarket schemes, aimed at reducing food waste by selling surplus food close to its expiry date, for a low price, to acquire food for himself and his partner. As Participant 03 points out, there is no choice around the items the recipients receives and therefore, resources such as cooking skills and equipment (freezer) may be required:

*‘Morrisons do this thing, I can't remember the exact name for it, but they have an app where you can buy parcels of things that are on their last legs, like Too Good To Go, I think it's called, erm, and they give you just a big box of loads of stuff that's maybe on its last couple of days of use … you kind of get what you get. But if you're able to cook, if you're able to have storage to freeze and refrigerate stuff then you should be fine’* (Participant 03, male, formula feeding, family of three)

### Theme 4

#### Don’t ask, don’t tell: limited to no conversations about finances or formula with health professionals

When asked about conversations around finances and formula with healthcare professionals (that could help ensure new parents are accessing all the services and relevant support to which they are entitled), participants overwhelmingly described a sense of don’t ask, don’t tell, where healthcare professionals appeared uncomfortable having such discussions. From our participants’ perspective, it appeared healthcare professionals preferred not to engage in these topics with new parents. Only one participant described such conversations which resulted in their health visitor helping them access support from a local food bank when they were struggling to afford food for themselves. Additionally, parents described difficult conversations with healthcare professionals surrounding infant formula, with participants indicating that they felt health professionals perceived such conversations as being seen to discourage breastfeeding. Participants described a journey of trial and error when it came to finding a suitable formula for their baby, often guided by information obtained from unregulated sources such as social media.

#### Almost no discussion around financial circumstances

Almost none of the participants interviewed could recall a discussion around their financial circumstances with any healthcare professionals such as their GP, Midwife or Health Visitor. However, at the same time, Participant 02, a combi feeding mum whose baby had allergies that required her to eat a dairy free diet, something she described as expensive, increasing the families food bills, spoke of her belief that there was little healthcare professionals could do to help unless someone was in extreme financial difficulty:

*‘They never asked... And I think, but even if, I'd of, let's just say they had asked...I don't think, what they would suggest? like I know you can get a payment from the social work to support you if you're kind of financial destitute but that obviously comes with stigma attached to it as well, erm, that wouldn't be a regular payment, so I don't know’* (Participant 02, female, combi feeding, family of four)

Others described conversations with health professionals as focusing on *‘how's your mental health and are you safe in your home’* (Participant 10, formula feeding, family of three) or problem and solution focused *‘it was more kind of…if baby has this problem, this is maybe a solution…for example, if they have reflux, you can use Gaviscon’* (Participant 03, male, formula feeding, family of three). However, Participant 03 went on to state his frustration that details, arguably the most important details for those living on a low income, were not shared, potentially due to assumptions made by health professionals on a family’s financial position:

*‘but they didn't say where you can get them [medicines for reflux] from or how expensive they are, which are the kind of key pieces of information you want to know… I don't know, I mean, we all like to make assumptions, it's human nature so they just thought, oh, you guys should be fine, I don't know, cause I don't know the scope of other families that they deal with’*

#### Strained conversations around infant formula

Parents described strained conversations with health professionals surrounding formula feeding. Reflecting on her interactions with health visitors and midwives, Participant 07 described sensing uncertainty from healthcare professionals as to whether they should be discussing infant formula at all with new mums. Participant 07 stated she understood that breastfeeding was prioritised, resulting in these strained conversations, but also expressed how these interactions left her feeling frustrated:

*‘It was quite funny because they, they were saying something about, you know, the formula, they would start with, I should, I'm not allowed to say it but…I think they are troubled...I do understand historically why, why they had to do it but I think it's just went a little bit too far’* (Participant 07, female, breastfeeding, family of three)

Participant 01, a single mother who reported struggling to find a suitable brand of formula for her baby, described how *‘if I mention formula, it kind of just gets shut down’* and that the *‘conversation changes pretty quickly’* whenever she tried to discuss this topic with her health visitor.

#### Trial and error and sources of advice on suitable formula

Throughout the data there is a sense of parents embarking on an often lengthy, and potentially unnecessary and costly journey of trial and error in finding suitable infant formula for their baby. For example, Participant 01 described trying out a range of different brands before settling on the one they felt best met their baby’s needs.

*‘It was Aldi's, then we tried Cow and Gate...then we moved onto SMA comfort formula for colic and constipation, which made an ever so slight difference, but he was still screaming in agony for hours, and then last week, I went onto to SMA soya formula’* (Participant 01, female, formula feeding, single parent)

The strained or limited conversations with healthcare professionals about formula and finances appears to have led some parents to seek information on infant formula from unregulated sources including internet sites such as YouTube and social media platforms including Facebook and TikTok.

*‘so we're on Kendamil…it's becoming a big thing because a lot of like YouTubers and TikTok'ers, like influencers, are using it, so people are using it as well and it's leaving short stock’*Participant 04 (female, formula feeding, single parent)

## Objective 2: Exploring parents experiences of realising (or otherwise) their feeding intentions during their infant’s first 6 months

### Theme 5

#### Breastfeeding: Positive views about breastfeeding but challenges dictate practice in reality

More than half of participants stated they had intended to breastfeed their baby due to the associated health benefits, however, in practice many parents struggled to feed their baby in line with their intentions. Reasons for not feeding in line with intention included lack of support, anxiety around milk production and baby not getting sufficient milk.

#### Best for health, bonding and budget friendly, the perceived benefits of breastfeeding

New parents living on a low income shared that their initial intention to breastfeed their baby was driven by their knowledge of the physical health benefits of breastfeeding for both mother and baby as well as emotional benefits in terms of bonding. Participant 02, a combi feeding mum, also spoke about the ease of breastfeeding in contrast to formula feeding:

*‘my reasons for breastfeeding were health for the baby, health for me, bonding, the whole of being like, you know, you breastfed your child and ease, definitely was one of the big reasons as well, cause it's just so easy, you don’t need to mess about, I know from doing a bit of the formula feeding with the prescribed formula that it was a faff’* (Participant 02, female, combi feeding, family of 4)

Furthermore, breastfeeding was also often perceived as cost saving or free:

*‘breastfeeding’s on demand, it's free, the right temperature, you don't need to take anything with you’* (Participant 07, female, breastfeeding, family of 3)

#### Didn’t go as planned…

Despite over half of participants stating they intended to breast feed, only one reported exclusively breastfeeding with two combi feeding their baby. Participants described breastfeeding as challenging and an anxiety provoking experience, as demonstrated in the following quotation:

*‘I tried in hospital, and I started getting really anxious really quickly that she wasn't getting enough, and the nurses had told me, no, you're fine... I couldn't let go of that idea that she wouldn't be getting enough from me…So, I asked if I could try pumping, and I got a pump given by one of the nurses, nothing came out but, again, I think I was spiraling, thinking oh my god, I can't, I need to formula feed…’* (Participant 06, female, formula feeding, family of 3)

Even those participants who breastfed or combi-fed their baby and reported good support from health professionals, acknowledged the challenges. Potentially reflecting societal views on breastfeeding, Participant 07 stated this ‘natural’ behaviour can be stressful and difficult for new mums to undertake:

*‘it's natural to, to breastfeed your baby but it's not actually coming so easy for every, for everyone…I don't think I had the, the, the, depression after birth or nothing like that but every day there was a, a few times a day when I would be just sit in the bathroom and crying because I can't feed her, she's screaming, I don't know what else to do and I think it's understandable that a lot of mothers will be, er, forced to give up’* (Participant 07, female, breastfeeding, family of 3)

## Objective 3: Investigating parents’ perceptions of factors that help or hinder their infant feeding experiences

### Theme 6

#### Income shortfall struggles: the provision of instrumental support from family and third sector organisations

All participants spoke about the instrumental support they received from family members (a parent, grandparent or sibling) and/or third sector organisations in alleviating their financial shortfalls. This instrumental support was provided in different ways including the use of the Cash First approach designed to reduce the need for the use of food banks and provide a more dignified response to support those in need of help.

#### Covering costs, the role of family

Participants described the generosity of family members in providing them with monetary assistance or helping them secure food during times of need, as demonstrated in the following quote from Participant 05, a single mum in receipt of Government financial assistance:

*‘then there's, my mum, she's a great help to me as well if I'm ever, ever struggling. She'll either give me money that I'm need, whatever I'm needing, or she'll go and get what I need for me if I can't get’* (Participant 05, female, formula feeding, single parent)

#### Support in the community (third sector organisations)

Third sector organisations were described as a lifeline for many low income parents. Participant 01 who described struggling to pay her bills and feed herself and her baby, shared how a local baby bank helped her acquire essential items including bottles:

*‘the first round of bottles I got he didn't like and the second round of bottles I bought, he didn't like so by the third ones, these were just like ones that I managed to scrape together in a charity shop, and he did like them. Couldn't afford them cause they were the most expensive bottle on the planet, so I had to use [local baby bank] and they rocked up at my door with like ten bottles and saved the day…’* (Participant 01, female, formula feeding, single parent)

Two participants shared that they received financial aid through the Cash First response, a Scottish Government initiative that allows parents (through third sector organisations) to access money or vouchers that enable them to purchase what they need. Here, Participant 03 discusses receiving Cash First support through a community food initiative based in the North East of Scotland:

*‘[community food initiative] actually gave us some funding. They gave us £60, which then meant we could get four tubs [of formula], and that's lasted us about a month. So, maybe a tub or a week or so, give or take...’* (Participant 03, male, formula feeding, family of three)

### Theme 7

#### Parent experiences of stigma and guilt accessing and receiving support

During discussions almost all participants described purchasing second hand baby clothes or equipment (pram, bottles, changing mat), from charity shops or online selling sites or acquiring items from a baby bank. This method of acquiring the things they needed for their baby was viewed positively, a chance to reuse items that someone else’s child had outgrown and reduce the high costs associated with buying new items. In contrast, when it came to talk around accessing emergency food provision, participants conveyed a sense of stigma:

*‘it's like, like, I used to have a really good job...taking your newborn baby and son over to queue outside a food van, it's embarrassing but, I had to do it’* (Participant 09, female, formula feeding, single parent)

Participant 04, described how the stigma associated with using the food bank in their local area; the fear of being recognised when accessing this support by someone you know (potentially unavoidable in a small town), acted as a barrier to and hindered engagement with this service:

***‘****we've got a food bank as well now, but not a lot of people uses it because of like, if someone sees you going in there it's like, it'll be the talk of the town, like, that's how [name of town] is, cause it's a small town…’* (Participant 04, female, formula feeding, single parent)

Parents also conveyed a sense of guilt around receiving support from family members and a reluctance to ask for help. The following quote from Participant 08 suggests the guilt she experienced, underpinned by her knowledge of the high cost of even basic equipment required for feeding a baby:

*‘so my mum and dad helped quite a lot out…And my granddad as well, he got, got us a couple of things…But I kind of feel like I couldn't ask for them so I kind of waited until like the last minute and they kind of offered…Especially cause like things are, you know, you've got, things are not cheap...Even like a sterilizers £15, £20 nowadays, you know, for like a, a basic one’* (Participant 08, female, formula feeding, family of three)

## Discussion

New parents in our study described significant challenges in feeding both their baby and themselves, often driven by economic and social factors. Parents spoke about the high cost of infant formula and healthy food items during a period of reduced income and increased outgoings and sacrificing their own nutritional needs to ensure their baby was adequately fed. While the majority of new parents recognised the benefits of breastfeeding, including perceptions of the healthfulness and the cost saving nature of this feeding mode, and intend to breastfeed their baby, many found it hard to follow through on their intentions. New parents described relying on family members or third sector organisations to help with the cost of feeding their baby and themselves, however, certain types of support were described as stigmatising. Questions arise around new, food insecure parents’ nutritional status given the steps they take to maximise their budget in order to prioritise the nutritional needs of their baby.

Driven by sociopolitical factors, parents talked about the impact of their restricted income and the resulting struggle to feed themselves and their family. For some, a reduction in income occurred as a result of the move from full time employment to being in receipt of statutory maternity pay (SMP). Survey research, tracking the impact of the cost-of-living crisis on pregnant women and new mothers, revealed 73% of women who were pregnant or on maternity leave worried a lot about money [35]. While workplace maternity schemes can differ, a third of employers in the UK offer the statutory rate of 90% of the individual’s average weekly wage for six weeks then £187.18 or 90%, whichever is less, for the remaining 33 weeks [36, 37]. Enhanced maternity pay usually consists of full pay (ranging from six to 13 weeks), occasionally followed by half pay, before moving to the SMP rate for the remainder of the maternity leave period [37]. The rate of £188.17 per week is less than half the national minimum wage for a 35-h week and less than a third of women’s average full-time income [38].

Since the global financial crash in 2008, the UK Government have championed the need to reduce the national budget deficit, introducing ‘austerity measures’, resulting in cuts to the welfare system [39]. Around 40% of Universal Credit claimants in the UK are in work, topping up inadequate wages in the face of the rising cost of living [40]. However, due to the continued tightening of eligibility criteria, many more working families who find themselves struggling financially are not eligible for Government financial assistance as they earn just over the minimum income threshold [41].

In the current study, parents in such circumstances talked about not knowing where to turn to for economic support and feeling ignored, with one parent expressing a sense of being punished, despite having worked all their adult life. In the UK, around half of low-income, working age households not claiming benefits (either because their earnings are deemed too high, being unable to claim due to immigration status or eligible but just not claiming) reported being in arrears with household bills and over 70% reported going without essentials (i.e., adequate heating) or experiencing food insecurity [41]. It has been suggested that cash grants and free school meals, regardless of eligibility for financial assistance, might go some way to alleviate the pressure these families face [41]. For those reliant on Government financial assistance, benefits such as Universal Credit have been widely criticized for not covering outgoings and creating severe financial strain [42]. Government schemes such as Healthy Start and Best Start are designed to help support families on a low income access fruit, vegetables, milk and infant formula [18, 43]. However, payments are very rarely uprated and are insufficient to cover the cost of infant formula for parents who choose to formula feed their baby [21, 44, 45]. Front of pack health claims on infant formula such as ‘closest to breastmilk’, meaningless given breastmilk is impossible to recreate, can be misleading and compel parents to buy more expensive brands of formula for fear of not providing their baby with the highest quality product [46]. Perhaps unsurprisingly, formula milk has become one of the most commonly shoplifted items from retail and pharmacy stores in the UK [47, 48]. Against this government policy and economic backdrop, it is understandable to find that our participants spoke of the economic struggle to afford food and formula whilst being in receipt of government financial assistance.

Of significant concern to us was finding that, driven by their economic circumstances, our participants were sacrificing their own nutritional intake, reducing the quality and quantity of food they purchased and consumed, to ensure their baby was fed. This aligns with existing research that reports mothers accounts, of what may be becoming somewhat normalised coping strategies, such as buying less healthy food, eating smaller meals or going without food themselves to prioritise feeding their children [35, 49, 50]. This may be of concern to UK policy makers and politicians given there is an increased risk and noted trend in the increased incidence of low birthweight babies being born in the UK [51]. In addition to the concerns around the nutritional status of babies born to food insecure mothers, the short, medium and long term health and wellbeing of parents themselves should be a public health concern and requires further focus and attention, given the prevailing economic situation in the UK and the continued high cost of living (UK [52]).

Participants in the current study described undertaking time consuming, effortful behaviours when discussing their shopping practices which involved visiting multiple stores to acquire their baby’s food and other essential items (nappies, wipes) within their limited budget. Such behaviours may be driven by an extensive knowledge of the price of food, formula and other essentials across multiple stores and a need to shop where they can rather than where they want in order to maximise their limited budget and acquire all the products they need as identified in previous research involving people experiencing food insecurity [53]. Restrictions placed on an individual’s agency to purchase the food they would like to consume can take an emotional toll and have negative consequences for an individual’s mental wellbeing [53], something that could be experienced more acutely by new parents, a potentially vulnerable period in an individual’s life [54]. Discussions with participants suggest that opportunities for intervention, i.e., signposting new parents to relevant support organisations or helping them access the financial support to which they are entitled, are potentially being missed.

Almost all new parents in the current study reported a lack of discussion around their financial circumstances with healthcare professionals such as their midwife, health visitor or GP. It is generally recognised that many physical and emotional health concerns stem from poverty and a lack of financial resources, however, it appears that patients financial circumstances are rarely discussed in primary care settings [55, 56]. Consequently, the need for this communication gap to be addressed within such environments, has been recognised [49, 55, 56]. NHS policy suggests that midwives and health visitors should be discussing families financial circumstances [43], however, interviews with participants in the current study indicated such discussions are not always taking place. A recent State of Health Visiting survey, involving health visitors from all four UK nations revealed, in 2023, 93% of health visitors reported witnessing a visible increase in poverty affecting families, i.e., an increase in the use of food banks, an increase in families skipping meals and the watering down of infant formula due to parents not having enough money to buy more [57].

Communication difficulties or gaps were also described by parents who tried to strike up conversations around infant formula with their health visitor or midwife. Participants in the current study conveyed a sense of midwives and health visitors as being almost prohibited from discussing formula feeding. This perspective aligns with findings of Lagan et al. [58], who suggested the strong focus on breastfeeding within the Baby Friendly Initiative, developed to help public services better support breastfeeding and adopted globally by Government, healthcare organisations, academic institutions and community services [59], prevented health professionals from freely and openly discussing formula feeding. One of the unintended consequences of this prevailing culture within early years health professionals was that our parents reported seeking advice from unregulated sources, for example social media sites. However, within the online space, it appears the International Code of Marketing of Breast-milk Substitutes, an internationally agreed voluntary code of practice which regulates the marketing of breastmilk substitutes to protect breastfeeding [60], is being disregarded by infant formula manufacturers [61]. Through social media channels, formula manufacturers have been found to be promoting false claims around the health benefits of added ingredients, the satiating effect of formula helping with sleep patterns and the need for specialised or ‘comfort’ products to solve conditions such as colic and reflux, despite evidence to the contrary [61]. On the other hand, a recent move to prevent social media influencers from being paid to discuss or endorse formula has been seen as having the potential to create a culture of fear around formula feeding, a move that could deprive parents of valuable insights and potentially further stigmatise parents who choose to formula feed [62]. All of this points to the need for healthcare professionals to incorporate discussions surrounding parents’ financial circumstances during routine appointments, as standard, to ensure parents receive all the financial support to which they are entitled. In addition, healthcare professionals should be willing to discuss the use of infant formula openly so that parents feel fully informed should they choose to use it, to try and reduce the need for parents to seek information from unregulated sources, e.g., social media and the internet.

The World Health Organisation (WHO) and The United Nations Children's Fund (UNICEF) recommend “exclusive breastfeeding, without any additional food or fluids, not even water, for the first six months”, with continued breastfeeding along with complimentary food for up to two years of age [63, 64]. Despite the health benefits of this behaviour, for both mother and baby, and for the continued health of the infant [65–68]. breastfeeding rates remain low in high income countries and are further reduced for those living in the most deprived quintiles [69–71]. Canadian studies have shown that while food insecure women initiate breast feeding at the same rates as food secure women, they are less likely to sustain exclusive breastfeeding [72]. Findings from the current study could be seen to support this. Participants acknowledged the health benefits of breastfeeding for both their baby and themselves, and the majority intended to breastfeed, however, less than half followed through with this intention. Feeding practices research indicates that food insecure mothers may struggle to meet breast feeding recommendations due to their experiences of insufficient breastmilk volume, latching difficulties, a lack of support for breastfeeding, a lack of safe spaces to breastfeed and a need to return to work [73–75]. While participants in the current study did not directly associate their decision not to breastfeed with their experiences of food insecurity, they shared some of the same worries (i.e., concerns around insufficient breastmilk and latching difficulties). The additional financial strain of acquiring good quality, nutritious food for themselves alongside such anxieties may make breastfeeding exceptionally challenging for those living on a low income. Going further, Frank [76] found that mothers experiencing food insecurity reported not only having difficulty maintaining the volume of milk they wanted to give their babies but worries that their breastmilk was of poor quality given the highly constrained quantity and quality of foods they were able to afford to consume. Some mothers shared that they felt compelled to switch to formula feeding due to fears they were risking their babies health by continuing to breast feed [76]. Participants demonstrated a good knowledge of the benefits of breastfeeding, including the perception of breastfeeding as being cost free. One participant estimated breastfeeding had saved their family £1,200 (the cost savings from not having to buy infant formula). However, this participant went on to describe how her infants dairy allergy meant she had to follow a dairy free diet, something that she admitted greatly increased her own food bills. Researchers in the United States and Canada argue that the financial costs associated with breastfeeding are substantial and do not represent a cost saving to the household budget when considered within the wider family context [77] and [78]. Mahoney et al. estimated that including equipment, vitamins, modified dietary requirements as well as time dedicated to feeding or expressing milk (4–6 h per day), breastfeeding could cost $8,640.07-$11,611.32, presenting a huge barrier for low income families [77]. Collectively, these findings demonstrate how upstream economic, social and environmental determinants might interact to influence infant feeding behaviour (breastfeeding or formula feeding), and potentially, health outcomes of the mother and infant.

Almost all participants spoke about receiving support from family members (parents, grandparents, a sibling), to help feed their baby and sometimes themselves. In line with existing research on coping strategies utilised by households experiencing food insecurity [79–81], participants described how family members would provide them with formula, food or money to enable them to purchase any items they might require. Equally, new parents in the current study talked about the support they received from third sector organisations including baby banks for equipment (pram, changing mat, baby bottles), that they would have otherwise struggled to acquire due to their limited budget. Baby banks have been described as helping to normalise the reuse of baby items, prevent unnecessary waste and build a community of support for parents (Baby Bank Alliance, n.d.). Discussions surrounding participants use of the baby banks were positive. This service was seen as environmentally and budget friendly and devoid of stigma. In contrast the receipt of emergency food provision through the use of food banks or food vans was described as highly stigmatising by participants. Accessing and receiving such support was accompanied by a sense of embarrassment and shame. This may have negative consequences for those parents who, by prioritising their infants’ nutritional requirements ahead of their own, have no money left to buy food for themselves. Utilising a food bank to procure food for their own consumption may provide those parents affected by infant food insecurity with food to eat (in theory at least) and help towards ensuring that the sacrifices they may feel forced to make to ensure their baby is fed, does not include having to go without food themselves. For others, acquiring food items from the food bank may allow them to stretch what limited income they have in order to afford to buy infant formula, nappies and other items they require. However, feeling compelled to use a food back for their own sustenance, or as a means of being able to afford infant formula, further exposes already vulnerable parents to the risk and mental health impacts of stigma and shame, synonymous with food bank use in general [80, 82–86]. Strategies such as the Scottish Government’s Cash-First plan, developed to address two of the main drivers of food insecurity in Scotland,insufficient and insecure incomes and reduce the need for food banks and the subsequent stigma attached to accessing food in this manner [25], could mitigate such experiences.

This study raises questions around the nutritional status of new parents experiencing food insecurity. Experiences of food insecurity and the reliance on the provision of support from family and third sector organisations, which may be inconsistent and stigmatising, could place significant mental stress on top of normal physical stressors associated with childbirth and the post-partum period. Further research is required to better understand whether visible increases in the numbers of families struggling to feed themselves and their infants, observed by midwives and health visitors [57], has increased discussions between healthcare professionals and new parents around families’ financial circumstances. If not, there is a need to understand why such conversations, that could help mitigate economic factors and subsequent behaviours, including diet and nutritional intake, which impact the health of parents and infants, are not being initiated. Poor nutritional patterns in the early years may lead to lifelong physical and mental health issues [87] and as such, should be a public health priority and the focus of targeted interventions and policies.

### Limitations

Despite extensive recruitment efforts and reassurance from our Advisory Group members as well as those working within different Government, third sector organisations and community groups who informed us that parents and carers attending services they provided were eligible due to their financial vulnerability, and would be willing to engage with the project, this proved not to be this case. Recruitment to the study was challenging, reflected in the small sample size (*N* = 11). While this potentially highlights the highly sensitive nature of the topic under investigation, it may also reflect a reluctance of parents to speak out about their struggle to feed their baby for fear of repercussion, for example, being reported to social services [49]. The research team attempted to engage with organisations within the local area that provide support for non-UK nationals, however, given time constraints of the project, such engagement was not achieved. Furthermore, all participants in the current study resided in Scotland. Conversations with a wide range of stakeholders in England during the recruitment period and our Advisory Group member based in Canada (a comparable high income country), indicated that our findings may be conceptually generalisable in this context. However, caution must be paid given the struggle with recruitment, the small sample size and the resulting lack of diversity of perspectives. Additionally, participants self-selected to the study,those parents struggling the most may have avoided taking part. More research is required to determine the full nature and extent of infant and maternal/parental food insecurity in the UK.

## Conclusion

New parents living on a low income experience many challenges in relation to feeding their baby and themselves. UK Government financial assistance, i.e., Universal Credit, Statutory Maternity Pay and schemes such as Healthy Start/Best Start often do not provide adequate financial support for new parents to allow them to ensure the nutritional requirements of their infants and themselves are fully met. Conversations between health professionals and new parents around families’ financial circumstances and infant formula are needed to help guarantee parents are accessing the financial support to which they are entitled and have the information required to make informed decisions around infant feeding. Parents living on a low income may require extra financial and instrumental support to help align their infant feeding practices with their initial feeding intentions. Services and third sector organisations should signpost or offer non-stigmatising, cash-first pathways to allow new parents to access financial assistance (money or vouchers), to help mitigate experiences of stigma associated with accessing certain types of support.

## Supplementary Information

Supplementary Material 1. Supplementary Data 1: Screening Questionnaire.

Supplementary Material 2. Supplementary Data 2: Interview Topic Guide.

## Data Availability

Due to the nature of the research, the data are not publicly available. Reasonable requests for access to the anonymised data that support the findings of this study will be considered by the corresponding author.

## Abbreviations

DIO: Diet and Health Inequalities
GP: General Practitioner
NHS: National Health Service
SERP: School Ethics Review Panel
SMP: Statutory Maternity Pay
SPF: Strategic Priorities Fund
TUKFS: Transforming the UK Food Systems
UK: United Kingdom
UNICEF: United Nations Children’s Fund
