# Supplementary material for: Navigating infant food insecurity: low-income parents infant feeding intentions and practices in the UK
Source: BMC Public Health. 2025 Dec 8;26:161. doi: 10.1186/s12889-025-25822-2 (PMC12797389; doi:10.1186/s12889-025-25822-2)

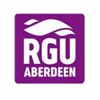

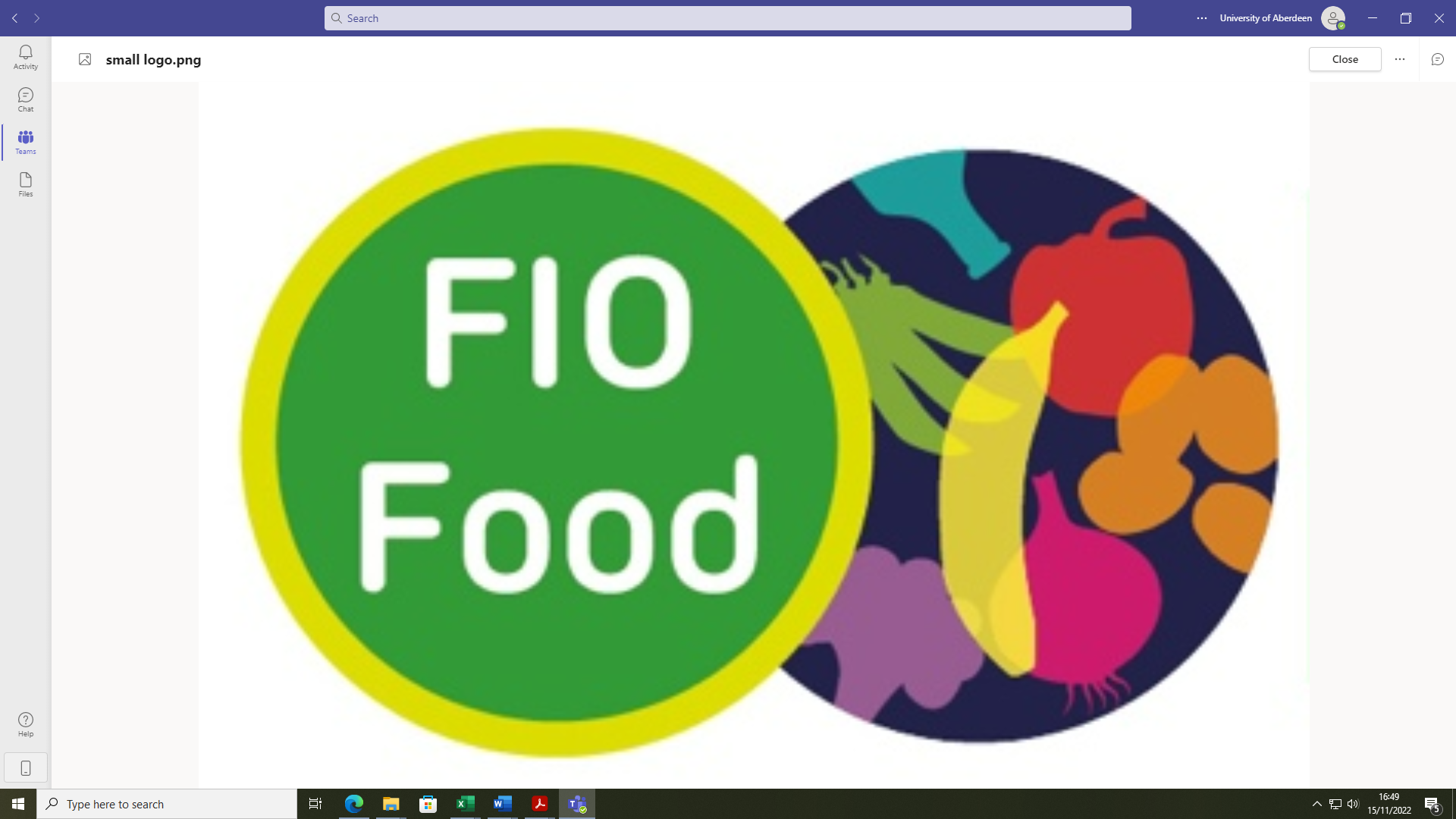


Dietary Health Inequalities (DIO) Food Project

Brief Screening Questionnaire

Thank you for taking an interest in being part of the DIO Food Project.

This questionnaire will help our researchers, Emma and Flora, find out if you are eligible to take part in one of our interviews. We will ask some questions about yourself, your baby and if you have experienced running out of food or have worried about running out of food.

You do not have to answer any question you don't want to answer, simply select 'prefer not to say' or skip the question.

All your answers are kept confidential, this means the questionnaire does not collect any information that could identify you. A file linking your name to your Participant ID code is stored safely and securely and can only be accessed by researchers from Robert Gordon University involved in the DIO Food Project. This helps to keep your answers to the questions in the questionnaire anonymous.

If you have anything you would like to ask before you complete this questionnaire, please email Emma at

[DIOFoodStudy@rgu.ac.uk](mailto:DIOFoodStudy@rgu.ac.uk)

Participant ID________

We would like to start by asking some questions about you. This information will help us ensure we are talking to a wide range of people about these important issues.

**1. Please tell us your age….**

| 16-24 | 25-34 | 35-44 | 45-54 | 55-64 | 65+ |
| --- | --- | --- | --- | --- | --- |
|  |  |  |  |  |  |

**2. With which gender do you identify:**

| Male | Female | Trans-gender man | Trans-gender woman | Non-binary | Prefer not to say | Other |
| --- | --- | --- | --- | --- | --- | --- |
|  |  |  |  |  |  |  |

If other (please specify)________________

**3. Please indicate your ethnicity:**

| **White…** |  | **Mixed/ Multiple ethnic groups…** |  |
| --- | --- | --- | --- |
| British |  | White and Black Caribbean |  |
| Irish |  | White and Black African |  |
| Other white background  (please specify)________________ |  | Other Mixed/ Multiple background  (please specify)________________ |  |
|  |  |  |  |
| **Black…** |  | **Asian/ Asian British…** |  |
| British |  | Indian |  |
| Caribbean |  | Pakistani |  |
| African |  | Bangladeshi |  |
| Other black background  (please specify)________________ |  | Chinese |  |
|  |  | Other Asian background  (please specify)________________ |  |

We want to hear from people with a range of different backgrounds including those who have moved here from other countries.

If you were not born in the UK, when did you most recently arrive to live here?

| Month | Year |
| --- | --- |
|  |  |

We are looking to talk to parents or care givers who are or have recently been primarily responsible for feeding an infant from birth to 6 months within their household.

**4. Have you been primarily responsible for feeding an infant (0-6 months)**

Yes

No

We would like to talk to parents or care givers who are or have been primarily responsible for feeding an infant from birth to 6 months during the last 2 years, therefore

**5. Was the infant you were responsible for feeding born after January 2022**

Yes

No

We are interested in finding out about where in the UK our participants live. For this next question we only require the first part of your postcode, for some people this will be 3 digits, for others this will be 4.

**8. Please enter the first part of your postcode:** __________________________

Unfortunately, we are unable to provide translation services a spart of this study. To ensure anyone interested is able to take part in a one-to-one chat or a group discussion, we would like to check how well you understand the English language.

**9. How best would you describe your use and knowledge of the English language?**

| Fluent |  |
| --- | --- |
| Excellent |  |
| Very good |  |
| Good |  |
| Poor |  |
| Very poor |  |
|  |  |

Within the interviews and focus groups, we would like to speak with people who are experiencing food insecurity.

**13. Please indicate if you have experienced the following (select all that apply):**

|  | Within the past 12 months I/we worried whether our food would run out  before we got money to buy more |
| --- | --- |
|  | Within the past 12 months the food I/we bought just didn't last  and we didn't have money to get more |
|  | None of the above |

Our final questions explore your financial situation to give an indication around any financial issues our participants may or may not be facing.

**14. Thinking about your current financial situation, please indicate which best describes your household:**

|  | We have financial difficulties which makes it hard to budget from month to month |
| --- | --- |
|  | We get by alright |
|  | We have no financial difficulties |
|  | Prefer not to say |

We really appreciate you taking the time to answer our questions. Emma will be in touch shortly to discuss what happens next.

If you have any immediate questions or comments, please get in touch with Emma at

[DIOFoodStudy@rgu.ac.uk](mailto:DIOFoodStudy@rgu.ac.uk)


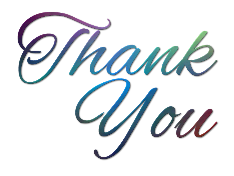

Supplement: Supplementary file 1 — Supplementary Material 1. Supplementary Data 1: Screening Questionnaire. [file 12889_2025_25822_MOESM1_ESM.docx]
