# Supplementary material for: Navigating infant food insecurity: low-income parents infant feeding intentions and practices in the UK
Source: BMC Public Health. 2025 Dec 8;26:161. doi: 10.1186/s12889-025-25822-2 (PMC12797389; doi:10.1186/s12889-025-25822-2)
Supplement: Supplementary file 2 — Supplementary Material 2. Supplementary Data 2: Interview Topic Guide. [file 12889_2025_25822_MOESM2_ESM.docx]

**Topic Guide Interviews DIO Food**

**Opening script**

**Introduction to the topic and instructions:**

Hello and welcome to our interview. Thank for agreeing to take part. My name is Emma and this is Flora and we’ll be going through the interview with you today. Before we start do you have any questions or concerns you’d like to raise that you thought of since you agreed to take part?

(Answer any questions)

Ok, great.

So, our research project is concerned with looking at parents and carers experiences of feeding an infant while living on a tight budget.

The interview should last about 30 minutes, but it depends how much or as little you feel you have to say about the things we will talk about today. You do not need to answer all the questions and if you don’t feel you have anything to say on a particular question, just let me know and I will move onto the next one. I’d also like to stress this not a test of knowledge (there are no wrong answers), we’re simply trying to find out your experiences and views on this topic and we are really grateful for the time you are giving and the insights you are sharing with us today. There will be time at the end where you can ask any questions you might have. We will be recording today’s discussion so we don’t miss anything important or have to write down everything you say. Your responses will be treated with full confidentiality, any potentially identifying information you share, such as names or places, will be removed so you can’t be identified. If you would like to take a break at any point, you can let me know and we can continue when you are ready. Just to let you know, you will be provided with a £25 shopping voucher as a thank you for taking part.

Do you have any questions before we start?

(Answer any questions)

**Interview Questions**

Before we get started, I wondered if you mind if I ask you a little bit more about yourself and your family?

*If the participant struggles to start this conversation use the following probing questions*

- **Can you tell me how old baby is?**
- **Were they born when they should have been or were they born early?**
  - **(If preterm ) Were they looked after in the neonatal unit or were they always with you?**
- **What is your experience of feeding your baby?**
  - **Could prompt: method (breastfeeding/ bottle feeding/ combination, feeding problems you needed help with? Who or what helped you?**
- **Have you any other children? How old are they?**
  - **If they have older children, ask how did you feed them and how did that go when they were a baby under 6 months?**
- **Do you have any worries about the health of your baby or children?**
- **How about your own health? How would you say your own health is just now?**

**For mothers:**

- **Is your health different since you had the baby?**
- **Did you have a caesarean section (and if so how did you recover?)**
- **Do you live alone or with a partner or spouse?**
- Depending on the age of the baby, ask if **partner or spouse was able to help you feed the baby or were they able to help you feed the baby when he or she was less than 6 months old? Or did someone else help (gran etc.) help out and was this feeding help important to them?**
- If the participant is not working at the present time, ask about **what they did for a living before they had their baby?**

**For father/ grandparent/ carer:**

- **Has your health changed since baby arrived?**
- **Do you live alone or with a partner or spouse?**
- Depending on the age of the baby, ask if **did you or were you able to help feed the baby when he or she was less than 6 months old? How was this experience?**
- If the participant is not working at the present time but stopped when the baby was born, ask about **what they did for a living before the baby arrived?**

You indicated before we started the interview that you had experienced some worries about putting food on the table for yourself or your family or had cut back on food for yourself to make it sure to feed your family/baby. Could you tell me a little about that experience?

[If the participant struggles to answer this question, ask about}

- **What caused them to start to worry about food,**
- **If it was a new thing for them or not?**
- **Are they still worrying about putting food on the table now?**
- **What helped them cope with putting food on the table, when they were struggling, or what helps them put food on the table if they are still struggling,**
- **Can you tell me about any strategies you use to make sure you have food for your baby?**

Some parents report cutting back their own food intake to try to make sure their children are fed. **Does this experience reflect your own experiences or not? *Ask*** *the participant to expand on their answer if they give a yes or no response with the question -* ***why is that the case for you?***

**Feeding intentions**

- Thinking back to when you were pregnant or at the point your baby was born, **can you recall how you planned to feed your baby, i.e., breast, bottle or a combination of the two?** **Can you recall why you planned to feed your baby that way? If breast – did finances play a part in your decision to breastfeed? If formula - was the cost of formula a factor in their decision of what milk to buy?**

**(if they had no plans, acknowledge that this is ok- if it feels appropriate, ask the following:**

- - - **Would you be comfortable sharing the reasons for not making plans around how you were going to feed your baby?)**
- **Would you say you were able to follow through on your plans or otherwise for as long as you planned to?**
- **If you were not able to do what you planned to do, why was that? If you were not able to follow through on your plans, did** **the food-related worries we talked about earlier, play any role in how you fed your baby, or not?**
- **If you were able to follow through on your plans, why do you think that is or was? Did any of the food-related worries we talked about earlier, play any role in your experiences and decisions about how you fed your baby, or not?**

**Feeding experiences**

- **Thinking about your experience of feeding your baby during this recent/Cost of Living crisis time, was there anything that you think might have made it easier for you to follow through on your plans or not?** *For example, probe for getting help to secure more money for you and your family during this time, getting more information and support about infant feeding from health professionals, the baby bank of other third sector organisation that were involved with at the time, having to return to work early due to money worries, no ability to breastfeed in the workplace or option to work from home?* [**If they had a baby previously probe for how previous feeding experiences may or may not have affected their feeding decisions this time?]**

**Final question**

**So I’ve reached the end of the questions I have here to ask you, but before we finish, is there anything you expected to be asked but I haven’t asked you, or you want to add to what we discussed during our interview so far?**
